# Recent Progress in the Research on RNA-Binding Proteins in Bone Development and Diseases

**DOI:** 10.3390/ijms25147735

**Published:** 2024-07-15

**Authors:** Hafiz Muhammad Umer Farooq, Lihuizi Yang, Mengru Cao, Zhihao Chen, Airong Qian, Kai Dang

**Affiliations:** Laboratory for Bone Metabolism, Xi’an Key Laboratory of Special Medicine and Health Engineering, Key Laboratory for Space Biosciences and Biotechnology, Research Center for Special Medicine and Health Systems Engineering, NPU-UAB Joint Laboratory for Bone Metabolism, School of Life Sciences, Northwestern Polytechnical University, Xi’an 710072, China; farooq@mail.nwpu.edu.cn (H.M.U.F.); yanglihuizi@mail.nwpu.edu.cn (L.Y.); chenzhihao@nwpu.edu.cn (Z.C.)

**Keywords:** RNA-binding proteins, osteoblastogenesis, osteoclastogenesis, osteoporosis, osteoarthritis

## Abstract

RNA-binding proteins (RBPs), which regulate gene expression through post-transcriptional modifications of RNAs, play a role in diverse biological processes that include bone cell development and bone tissue formation. RBP dysregulation may result in aberrant bone homeostasis and contribute to various bone diseases. The function of RBPs in bone physiology and pathophysiology and the underlying molecular mechanisms have been extensively studied in recent years. This article provides a review of such studies, highlighting the potential of RBPs as pivotal targets for therapeutic intervention.

## 1. Introduction

Bones are a fundamental component of the musculoskeletal system that enable locomotion and movement and support and protect vital organs. Following injury, bone repair and regeneration is critical for restoring the full functionality of bones. This restoration process is controlled by mechanisms that regulate the balance between the formation and activity of bone-forming osteoblasts and bone-resorbing osteoclasts within the bone microenvironment [1]. Bone-marrow-derived mesenchymal stem cells (MSCs) are progenitor cells that can differentiate into osteoblast lineage cells to facilitate osteogenesis upon injury [2,3]. The commitment of osteoblast lineage cells to osteoprogenitor cells and subsequent preosteoblasts is dependent on runt-related transcription factor 2 (RUNX2) and SRY-box transcription factor-9. The induction of osterix (OSX) by Wnt-β promotes the maturation of osteoblasts [4,5]. Mature osteoblasts can incorporate into the bone matrix and undergo terminal differentiation to form osteocytes, the most abundant and long-lived cells in bones [6]. Osteocytes are the master regulators of bone formation induced by mechanical stimulation, as well as bone loss caused by disuse. The receptor activator of nuclear factor-kappa B ligand (RANKL) secreted by osteocytes is the most important factor for osteoclast-mediated bone resorption under pathological conditions [7,8].

RNA-binding proteins (RBPs) bind to RNA molecules to regulate RNA splicing, localization, stability, and translation [9,10]. Disruption in RNA-RBP networks can contribute to the development of many pathological conditions [11]. The function of RBPs in bone physiology and pathophysiology and the underlying molecular mechanisms have been extensively studied in recent years. For example, RBPs can regulate osteogenic differentiation of bone-marrow-derived MSCs and abnormal bone metabolism in osteoporosis [12,13]. This article provides a review of recent progress in the research on RBPs in bone development and diseases and highlights the potential of RBPs as targets for therapeutic intervention.

## 2. RBPs Involved in the Differentiation of Bone-Marrow-Derived MSCs

The bone marrow contains a multipotent and heterogeneous population of MSCs that are considered committed progenitors for bone cell formation [14]. Dysregulation in the differentiation of these cells can lead to bone diseases [15]. Recent evidence has indicated that RBPs could influence the differentiation of bone-marrow-derived MSCs through the molecular mechanisms elaborated below.

### 2.1. Musashi RBPs

The Musashi RBPs, which comprise two structurally similar proteins Musashi-1 and Musashi-2, were first identified in a 1994 study on sensory organ development in Drosophila [16]. During germ cell development, Musashi-1 is mainly localized in the cytoplasm of mitotic gonocytes and spermatogonia, while Musashi-2 is predominantly localized in the nucleus of meiotic spermatocytes and differentiating spermatids [17]. A subsequent study found that Musashi-1 can translocate to the nucleus to bind the transcriptionally silenced XY chromatin domain in meiotic pachytene spermatocytes, resulting in the release of Musashi-1 RNA-binding targets [18]. This supports Musashi-1 as a key regulator of post-transcriptional control during early spermatogenesis.

Padial-Molina et al. [19] investigated the nuclear and cytoplasmic expression of Musashi-1 in different cell types involved in bone formation in an experimental model of rat femur bone fracture. Osteoblasts demonstrated both nuclear and cytoplasmic Musashi-1 expression, which was absent in mature chondrocytes. Over a 14-day period of callus formation, there was a gradual increase in Musashi-1 expression in MSCs, osteoblasts, and osteocytes as compared to the control. After 14 days of healing, Musashi-1 was detected at a much higher level in bone fracture callus as compared to fibrous connective tissues of the unfractured bone. In addition, Musashi-1 was predominantly found in the nucleus of MSCs and immature chondrocytes of the fibrocartilage callus. RUNX2 and POSTN (periostin) followed an almost identical expression pattern to that of Musashi-1 in MSCs, osteoblasts, and chondrocytes, suggesting an interrelated function between Musashi-1, RUNX-2, and POSTN during the bone healing process.

O’Valle et al. [20] detected high Musashi-1 levels in the nucleus and cytoplasm of MSCs derived from bone grafts after maxillary sinus surgery in humans. Musashi-1 was also found in the nucleus and cytoplasm of osteoclasts and osteoblasts, but it was exclusively localized in the nucleus of osteocytes. No Musashi-1 expression was detected in adipocytes. Although RUNX2 expression remained unchanged in both native and grafted bone, POSTN expression was increased in grafted bone and correlated with the expression of Musashi-1, suggesting an interrelated function of the two genes to regulate osteogenesis during bone regeneration.

Padial-Molina et al. [21] characterized the expression of Musashi-1 during the osteogenic differentiation of MSCs from the oral cavity, in relation to RUNX2. MSCs derived from dental pulp exhibited the highest expression of cell stemness markers such as *MYC*, *SOX2*, *POU5F1*, and *NANOG* after xenografting. An investigation of Musashi-1 mRNA expression in MSCs of different origins showed that Musashi-1 was significantly elevated in MSCs from the dental follicle but not from the alveolar bone or tooth germ after 14 days of differentiation. After 28 days of differentiation, Musashi-1 was detected in MSCs from grafted bone. Similar to the findings of O’Valle et al. [21], POSTN expression was increased in grafted bone, while RUNX2 expression remained unchanged.

Suo et al. [22] detected a gradual increase in Musashi-2 expression during the differentiation of bone-marrow-derived MSCs into osteoblasts. However, Musashi-2 expression gradually decreased following the induction of adipocyte differentiation. These findings indicate that Musashi-2 may play a vital role in determining the differentiation fate of bone-marrow-derived MSCs towards either osteoblasts or adipocytes. Musashi-2 was detected in the growth plate, periosteum, and cancellous bone but not in compact bone. Musashi-2 knockdown resulted in significant reductions in trabecular bone thickness and bone mass and a markedly shortened femur infiltrated with adipocytes. Bone-marrow-derived MSCs lacking Musashi-2 showed significantly -decreased expression of *Col1α1*, *Atf4*, *Alp*, and *Bsp* and increased expression of *Cebpα* and *Cebpβ* during osteogenic and adipogenic differentiation, respectively. RNA sequencing techniques confirmed these results, suggesting that the Musashi-2 knockout might promote osteoblast differentiation and suppress adipogenic differentiation. Musashi-2 overexpressed in bone-marrow-derived MSCs was found to bind to the peroxisome proliferator-activated receptor γ (PPARγ) mRNA, promoting the differentiation into osteoblast lineage cells, whereas the enrichment of Cebpα transcript by Musashi-2 influenced adipocyte differentiation from MSCs.

### 2.2. ZFP36L1

**Figure 1:**
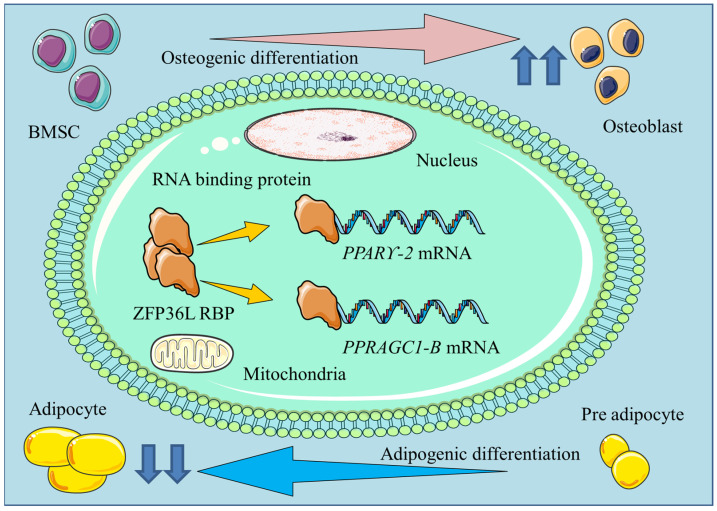
Schematic diagram illustrating the mechanisms by which ZFP36L-1 regulates the osteogenic and adipogenic differentiation of bone-marrow-derived MSCs in the bone microenvironment. ZFP36L-1 binds to the PPARγ and PPRAGC1-B mRNA in the cytoplasm of differentiating MSCs to promote or suppress osteogenic and adipogenic differentiation.

Liu et al. [23] investigated the role of the RBP ZFP36L1 in adipogenic differentiation of bone-marrow-derived MSCs in aplastic anemia (AA) in the presence of levamisole. Levamisole is an adipogenic differentiation suppressor that acts through improving the bone microenvironment. MSCs from AA patients exhibited increased expression of adipogenic markers such as PPARγ, PLIN1, LPL, and FABP4, along with a greater number of lipid droplets as compared to controls. The induction of adipogenic differentiation led to decreased ZFP36L1 expression in AA MSCs, and levamisole treatment restored ZFP36L1 expression. ZFP36L1 knockdown enhanced the expression of adipogenic differentiation markers *PPARγ*, *PLIN1*, *LPL*, and *FABP4* and increased lipid droplet formation in AA MSCs, while ZFP36L1 overexpression downregulated the adipogenic differentiation markers and decreased lipid droplet formation. Mechanistically, Liu et al. [23]. found that ZFP36L1 could downregulate PPRAGC1B to mediate the effects of levamisole. The associated mechanisms are shown in Figure 1.

### 2.3. PUM RBPs

The human PUF family proteins, PUM1 and PUM2, are RBPs that bind to a PUM recognition element (PRE) in the 3′-UTR of target mRNAs. Lee et al. [24] reported that PUM2 can bind to the 3′UTR region of the *JAK2* and *RUNX2* mRNA to mediate the crosstalk between osteogenic and adipogenic differentiation. PUM2 overexpression downregulated JAK2 and RUNX2, but it did not affect the colony-forming ability of bone-marrow-derived MSCs. Instead, PUM2 overexpression induced PPARγ and ALP expression and promoted adipogenic differentiation. CRISPR/CAS-9-mediated PUM2 silencing prevented fat accumulation and enhanced bone formation in zebrafish embryos.

Yoon et al. [25] detected reduced PUM1 expression during MSC senescence. PUM1 modulated the activity of nuclear factor κβ (NF-κB) by post-transcriptionally targeting the mRNA translation of Toll-like receptor 4 (TRL4). PUM1 overexpression rescued the proliferation and colony-forming ability of MSCs treated with H_2_O_2_. Additionally, PUM1 was found to be downregulated in damaged cartilage tissues from patients with osteoarthritis (OA). Injections of a lentiviral vector carrying PUM-1 protected the knee cartilage integrity in a mouse model of medial meniscus damage-induced osteoarthritis.

### 2.4. ELAVL1

The RBP embryonic lethal abnormal version like-1 (ELAVL-1) was predominantly localized in the nucleus in MSCs undergoing osteogenic differentiation, where it promoted differentiation [26]. Selective ELAVL1 knockdown in mouse bone-marrow-derived MSCs altered alizarin red staining and the transcript levels of *RUNX2*, *Col1a1*, and *Fabp4* but not *OSX.* ELAVL1 silencing also reduced the expression of a number of genes involved in adipogenesis. RNA-sequencing of bone-marrow-derived MSCs during differentiation showed that ELAVL1 knockdown upregulated genes associated with extracellular matrix (ECM) formation and collagen synthesis at the transcriptional level. However, the effect of ELAVL1 overexpression on osteogenic and adipogenic differentiation was not evaluated.

## 3. RBPs Involved in Osteoblastogenesis

Bone remodeling commences with MGS differentiation to mononuclear osteoblasts, a process under the control of transcriptional factors such as RUNX2 [5]. Osteoblasts regulate bone metabolism, mineralization, and the synthesis of ECM proteins such as osteocalcin and alkaline phosphatase. In addition, osteoblast-derived signaling molecules facilitate intercellular interaction with osteoclasts. Osteoblast-derived RANKL plays a role in abnormal bone remodeling and mineralization in osteoarthritis [27,28]. Recent studies have indicated that RBPs are involved in the post-transcriptional regulation of osteoblastogenesis.

### 3.1. ZFP36L1

Tseng et al. [29] reported that the RBP ZFP36L1 promoted osteoblastogenesis but inhibited adipogenic differentiation. Lower mRNA levels of ZFP36L1 were detected in the femur and MSCs of aged mice compared to younger controls. ZFP36L1 knockdown in MC3T3-E1 preosteoblasts inhibited their differentiation into osteoblasts, as evidenced by decreased levels of osteocalcin and osteopontin. However, ZFP36L1 knockdown upregulated adipogenic differentiation markers, including PPARγ-2, aP2, and adiponectin. ZFP36L1 overexpression in C3H10T1/2 cells enhanced osteoblastogenesis but reduced *PPARγ2* expression and decreased lipid droplet formation. These findings support the binding of ZFP36L1 to the PPARγ-2 mRNA as a key factor directing osteoblast and adipocyte lineage differentiation.

### 3.2. BICC1

Mesner et al. [30] investigated the associations of the RBP Bicaudal C homolog-1 (BICC1) with quantitative trait loci related to osteoblastogenesis. The results identified BICC1 as a genetic determinant of osteoblastogenesis and bone mineral density. Linkage data analysis identified *Bmd43* and *Bmd42* as regulators of bone mineral density. BICC1 was found to be localized in close proximity to *Bmd43*, and the two proteins showed a correlated expression. The absence of BICC1 was associated with reduced bone mineral density in the femoral bones of mice, but the bone in the medullary region was not altered. In addition, a correlation was observed between trabecular bone volume and bone fraction volume in BICC1 knockdown mice. High BICC1 expression was detected in primary calvarial osteoblasts during differentiation, with relatively lower levels observed at the midpoint of differentiation. siRNA-mediated BICC1 knockdown results indicated that BICC1 regulated the activity of alkaline phosphatase and the expression of osteogenic markers during bone formation. BICC1 was found to target polycystic kidney disease 2 (PKD2) to inhibit osteoblastogenesis. BICC1 knockdown reduced alizarin activity, which was rescued by *PKD2* overexpression.

### 3.3. RBM3

Kim et al. [31] reported that the RBP RBM3 facilitates osteoblast differentiation through extracellular-signal-regulated kinase (ERK) phosphorylation. RBM3 was expressed during the osteoblastic differentiation of MC3T3E1 cells. RBM3 overexpression upregulated early osteogenic differentiation markers such as RUNX2 and osteocalcin, while RBM3 knockdown decreased the expression of RUNX2. Conversely, RUNX2 knockdown did not affect RBM3 expression. RBM3 overexpression enhanced the phosphorylation of ERK and p38 kinase (p38) but suppressed the phosphorylation of c-Jun N-terminal kinase (JNK).

### 3.4. Sam68

Richard et al. [32] found that the elimination of the RBP Sam68 prevented bone loss in aging mice. Sam68 was highly expressed in the metaphysis of long bones and cartilage, and it was localized in the nucleus of proliferating osteoblasts. Mouse embryos deficient in Sam68 showed no significant developmental abnormalities—no tumor formation or immunogenic reactions. Sam68-deficient aging mice exhibited no cortical bone thinning or trabecular bone loss, and the bone mineral density of the femur and vertebrae showed significant improvement over control mice. In line with this, aging Sam68-deficient mice showed a distinct pattern of bone structure preservation, as evidenced by the results from ALP and TRAP staining and mineral deposition assessment.

Sun et al. [33] detected high Sam68 expression in the synovial tissue of rheumatoid arthritis (RA) patients, which was predominantly localized in fibroblast-like synoviocytes (FLS). TNFα stimulated Sam68 expression and induced its translocation from the cytoplasm to the nucleus. Sam68 knockdown in FLS suppressed TNFα, IL-6, and MMP-1 expression and inhibited cell proliferation, migration, and invasion. These findings underscore the important function of Sam68 in regulating FLS pathophysiology in RA in the presence of TNFα.

### 3.5. SAMD4

Niu et al. [34] found that the insertion of a PiggyBac transposon allele at the sterile alpha motif domain containing protein 4 (SAMD4) locus reduced the expression of the RBP SAMD4, resulting in a smaller body size and a shortened lifespan in mice. SAMD4 deficiency was associated with atypical skull development, decreased osteogenesis, and delayed ossification of the tibia. SAMD4-deficient mouse osteoblasts displayed decreased expression of osteoblast differentiation markers and ALP activity, highlighting the regulatory role of SAMD4 in osteoblastogenesis. Mechanistically, SAMD4 was shown to bind to the mitogen-inducible gene 6 (MIG6) mRNA and impair its translation.

**Figure 2:**
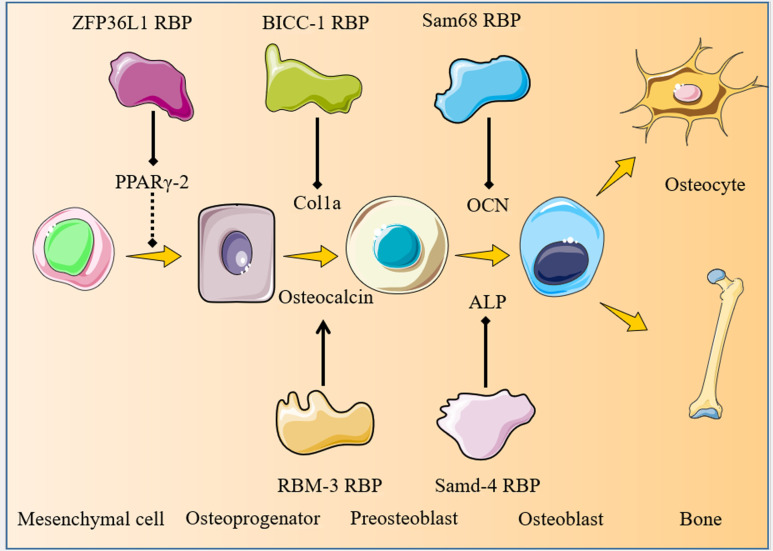
Schematic diagram illustrating the regulation of osteoblastogenesis by RBPs during bone development. ZFP36L1 RBP regulates PPARγ-2 expression during MSC commitment to osteoprogenitor cells. BICC1 and RBM3 modulate Col1a and osteocalcin expression during preosteoblast formation.

A schematic diagram illustrating the regulation of osteoblastogenesis by RBPs is presented in Figure 2.

## 4. RBPs Involved in Osteoclastogenesis

Monocyte colony stimulating factor and RANKL stimulate multinuclear osteoclast differentiation from the fusion of macrophage/monocyte precursors in the bone microenvironment. This process perpetuates a dynamic equilibrium of bone formation and resorption during bone remodeling. Excessive osteoclast activity can disrupt the bone microarchitecture and increase bone fragility, leading to osteoporosis or osteoarthritis. Here, we discuss the molecular mechanisms by which RBPs regulate osteoclastogenesis, as illustrated below.

### 4.1. CPEB RBPs

The CPEB family of RBPs, which include CPEB1, 2, 3, and 4, are widely expressed in warm-blooded mammals [35]. Arasaki et al. [36] detected elevated CPEB4 expression in RAW264.7 cells undergoing osteoclastic differentiation induced by RANKL. RANKL stimulated CPEB4 translocation from the cytoplasm to the nucleus through activation of the PI3K-Akt signaling pathway. CPEB4 deletion led to the decreased expression of osteoclastic differentiation markers such as *Acp5*, *Nfatc1*, *Ctsk*, and *RANK*. Yang et al. [37] found that CPEB4 was upregulated in metastatic tumors compared to the non-metastatic tumors of human osteosarcoma. CPEB4 overexpression increased M2-like polarization of activated RAW264.7 macrophages, as evidenced by enhanced CD206 and Arg1 expression. Supernatants from activated RAW264.7 macrophages overexpressing CPEB4 increased U2OS and MG-63 cell invasion and migration, highlighting the role of macrophage-derived CPEB4 in human osteosarcoma tumorigenesis. In addition, CPEB1 was implicated in the malignant progression of human osteosarcoma [38].

### 4.2. Musashi2

Fujiwara et al. [39] found that Musashi-2, but not Musashi-1, was significantly upregulated during RANKL-induced osteoclastic differentiation. Musashi-2 knockdown decreased the number of TRAP-positive cells and downregulated the osteoclast markers *Nfatc1*, *Ctsk*, and Acp5. The Western blotting results revealed that Musashi-2 promoted osteoclastogenesis through Numb-independent mechanisms.

### 4.3. Tristetraprolin

Mice deficient in the RBP tristetraprolin (TTP) exhibited increased alveolar bone loss over time, along with amplified inflammatory cell infiltration in the periodontal epithelium and maxillary tissues [40]. TTP-deficient maxillae showed an increased number of osteoclasts around the bone perimeter, and these osteoblasts were larger in size compared to wildtype control, and they exhibited increased resorptive function. Elevated levels of tumor necrosis factor-α (TNFα), a master modulator of osteoclastogenesis, were detected in the serum of TTP-deficient mice. It was postulated that TTP targets TNFα to suppress osteoclastogenesis and bone resorption.

### 4.4. IMP2

**Figure 3:**
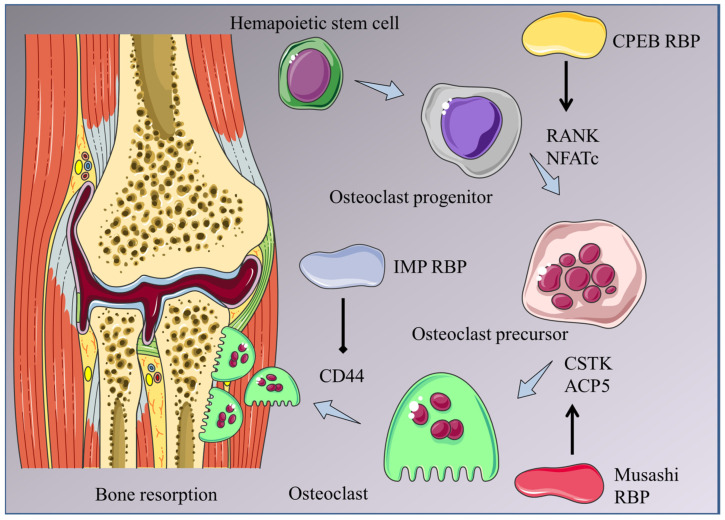
Schematic diagram illustrating the regulation of osteoclastogenesis by RBPs. Hematopoietic stem cells differentiate into osteoclast progenitor cells and osteoclast precursor cells, which eventually form mature osteoclasts. CPEB promotes RANK and NFATc expression during the formation of osteoclast progenitor cells. Musashi interacts with CSTK and ACP5 to promote the formation of mature osteoclasts from osteoclast precursor cells.

Liu et al. [41] demonstrated that IMP2, an insulin-like growth factor 2 mRNA-binding protein, can regulate osteoclast function and adhesion. IMP2-deficit mice showed more immature bone structures and a thicker hypertrophic chondrocyte layer in the femoral metaphysis compared to wildtype mice. IMP2-deficient osteoclasts displayed impaired resorptive activity and reduced adhesion to the bone surface owing to defects in the CD44-osteopontin signaling pathway (Figure 3).

### 4.5. Mex3B

The RBP Mex3B can inhibit osteoclastogenesis by negatively regulating the level of the dendritic-cell-specific transmembrane protein (DC-STAMP) mRNA [42]. Mex3B-deficient mice exhibited reduced bone density, decreased trabecular bone volume, and enlarged osteoclasts in the tibia and femur. Bone-marrow-derived macrophages deficient in Mex3B exhibited increased DC-STAMP mRNA level and enhanced osteoclastic differentiation upon RANKL stimulation. In addition, Mex3B overexpression suppressed osteoclastic differentiation of RAW264.7 macrophages. The results from qPCR assays indicated that Mex3B regulated the transcription of the DC-STAMP gene or the stability of the nascent transcript.

### 4.6. QKI

The femoral metaphysis of mice lacking the RBP QKI showed decreased bone mineral density and cancellous bone formation [43]. QKI-deficient mice showed elevated levels of intraosseous inflammatory cytokines such as TNFα, a suppressor of osteoblastogenesis and a promoter of osteoclastogenesis. Mechanistically, QKI downregulation enhanced osteoclastogenesis by activating the MAPK and NF-κB signaling pathways. Rauwel et al. [44] investigated the role of QKI5 in HCMV-mediated osteoclastogenesis and bone resorption. HCMV infection upregulated QKI5 and induced its translocation from the cytoplasm to the nucleus to interact with the immediate early 2 viral gene, leading to the downregulation of the CSF-1F and RANK mRNA. QKI5 knockdown exacerbated HCMV-mediated osteoclastogenesis, while QKI5 overexpression inhibited osteoclastogenesis and protected against bone destruction in a mouse model of calvarial bone erosion.

## 5. RBPs Associated with Bone Diseases

Bone diseases such as osteoporosis, osteosarcoma, and osteoarthritis are quite common, but their etiology is not fully understood. Identifying new target molecules for therapeutic intervention is critical for improving the clinical outcome of patients with bone diseases.

### 5.1. RBPs Associated with Diabetic Osteoporosis

Diabetic osteoporosis is a life-limiting complication of diabetes mellitus (DM). Epidemiological data show that DM is a major risk factor for osteoporotic fracture [45]. High glucose suppresses osteocalcin expression to inhibit osteoblast differentiation and bone formation, contributing to diabetic osteopenia [46]. A number of RBPs have been implicated in the pathogenesis of diabetic osteoporosis as illustrated below.

#### 5.1.1. ELAVL1

Ren et al. [47] detected elevated levels of ELAVL1 and divalent metal transporter 1 (DMT1) in the femur of DM mice. ELAVL1 knockdown in DM mice increased osteogenesis and prevented bone loss. In addition, ELAVL1 knockdown in MC3T3-E1 preosteoblasts enhanced osteoblastic differentiation and mineralization. Mechanistic studies suggest that ELAVL1 may function through regulating DMT1.

#### 5.1.2. PCBP1

Ma et al. [48] found that high glucose upregulated the RBP PCBP1, reduced the viability of hFOB1.19 osteoblasts, and caused mitochondria atrophy. PCBP1 overexpression enhanced OPG and OCN expression and increased calcium deposition, while PCBP1 knockdown showed opposite effects. Mechanistically, PCBP1 may function through suppressing ferroptosis upon high glucose stimulation.

### 5.2. RBPs Associated with Osteosarcoma

Osteosarcoma is a common bone disease, with an estimated incidence rate of 9 per million individuals. Due to the lack of diagnostic tests and limited treatment options, the survival rate and prognosis of osteosarcoma patients are extremely poor. A number of RBPs have been implicated in the pathogenesis of osteosarcoma as illustrated below.

#### 5.2.1. HuR

Liu et al. [49] detected high expression of the RBP HuR in stage T2 osteosarcoma (OS) tissues and various OS cell lines, including MG63, SAOS2, U2OS, and SJSA-1. HuR knockdown decreased SJSA-1 cell proliferation, migration, and epithelial–mesenchymal transition (EMT). Mechanistically, HuR may function through targeting AGO2. Similarly, Pan et al. [50] showed that HuR was highly expressed in human OS tissues, and HuR knockdown decreased MG63 OS cell viability and EMT. Mechanistic investigation suggested that HuR may promote OS progression by disrupting the interaction between miR-142-3p and HMGA-1. Xu et al. [51] reported that HuR knockdown repressed SAOS2 OS cell migration, invasion, and stemness through inhibiting YAP.

#### 5.2.2. LIN28A

Wang et al. [52] detected high expression of the RBP LIN28A in human OS tissues compared to adjacent tissues. LIN28A knockdown in MG63 OS cells decreased cell viability and migration and increased apoptosis. LIN28A was found to associate with the long noncoding RNA (lncRNA) MALAT1 and increase its stability and expression.

#### 5.2.3. PTBP-1

Zhou et al. [53] reported that the LncRNA ZMIZ1-AS1 was upregulated in human OS tissues, which was controlled by the transcription of SOX-2 and MYC in response to the hippo signal pathway. ZMIZ1-AS1 promoted OS cell proliferation, migration, and invasion by recruiting the RBP PTBP1 to stabilize the ZMIZ1 mRNA. PTBP1 or ZMIZ1 overexpression rescued the suppressive effects of ZMIZ1-AS1 knockdown on OS cellular processes.

### 5.3. RBPs Associated with Osteoarthritis

#### 5.3.1. ZFP36L1

Son et al. [54] found that the RBP ZFP36L1 was upregulated in human and mouse osteoarthritis (OA) chondrocytes and cartilage. Although ZFP36L1 overexpression alone in mouse knee-joint tissue did not modulate OA symptoms, ZFP36L1 silencing significantly reduced cartilage destruction and osteophyte formation in OA mice. Mechanistically, ZFP36L1 may function through directly targeting members of the heat shock protein 70 family such as HSPA1A and HSPA1B. HSPA1A overexpression reduced OA symptoms after surgical destabilization of the medial meniscus.

#### 5.3.2. GNL3

The RBP GNL3 was found to be upregulated in the synovial tissue and fluid of OA patients [55]. RNA sequencing revealed elevated GNL3, PTN, and IL-24 levels in human OA lesions. GNL3 knockdown studies identified IL-24 and PTN as target genes for GNL3 [56].

#### 5.3.3. TDP-43

Chang et al. [57] found that the RBP TDP-43 and its binding partner G3BP1 were downregulated in the degenerated cartilage of OA patients. TDP-43 concentration in human OA synovial fluid was positively correlated with IL-1β. In addition, an intra-articular injection of recombinant TDP-43 improved cartilage degradation and subchondral bone remodeling in a rat model of post-traumatic knee OA.

#### 5.3.4. SND1

Lv et al. [58] found that an IL-1β treatment of rat primary chondrocytes upregulated the RBP SND1 and induced ferroptosis. SND1 knockdown suppressed chondrocyte ferroptosis induced by IL-1β, which could be abolished by HSPA5 knockdown. In a rat model of OA with surgical destabilization of the medial meniscus, SND1 knockdown upregulated HSPA5, suppressed ferroptosis, and alleviated cartilage tissue damage. Mechanistically, SND1 bound to the 3′UTR of the HSPA5 mRNA and destabilized the HSPA5 mRNA.

### 5.4. RBPs Associated with Osteoporosis

#### 5.4.1. PTBP1

Yu et al. [59] reported that the LncRNA SNHGI interacted with the RBP PTBP1 to upregulate DNMT1, which, in turn, suppressed osteogenic differentiation of bone-marrow-derived MSCs and contributed to osteoporosis.

#### 5.4.2. HuR

Liu et al. [60] reported that HuR was downregulated in the bone tissue of ovariectomized (OVX) mice. HuR silencing suppressed osteoblastic differentiation of MC3T3-E1 preosteoblasts, as evidenced by decreased Runx2 and Osterix expression and reduced ALP activity. Mechanistically, HuR stabilized the LRP6 mRNA and promoted its translation through binding to the 3′UTR of the LRP mRNA, leading to the activation of the Wnt pathway. In OVX mice, injection of HuR-overexpressing bone-marrow-derived MSCs alleviated OA symptoms and enhanced femoral bone formation. Chen et al. [61] found that the circular RNA circStage-1 was downregulated in human and rat osteoporotic bone tissues. CircStag-1 overexpression promoted osteogenic differentiation of bone-marrow-derived MSCs. Mechanistically, circStag1 interacted with HuR and promoted its translocation from the nucleus to the cytoplasm, where HuR stabilized and enhanced LRP5/6 and β-catenin, thereby activating the Wnt pathway and stimulating osteogenic differentiation.

### 5.5. RBPs Associated with Calcific Tendinopathy

#### RBFOX2

Cho et al. [62] found that the RBP RBFOX2 was localized in the cytoplasm in calcific tendons and the nucleus in normal tendons of human subscapularis. RBFOX2 regulated CHD2 and MBNL1 mRNA splicing in the nucleus. Thus, the cytoplasmic localization of RBFOX2 in calcific tendons may have affected overall mRNA splicing and gene expression, contributing to the development of calcific tendinopathy.

## 6. Summary and Perspectives

This review article discusses RBPs involved in bone development and bone-related diseases, along with their mechanisms of action. Through interacting with target RNA molecules, RBPs regulate the osteogenic differentiation of bone-marrow-derived MSCs and the formation of osteoclasts by the fusion and differentiation of macrophages, thereby modulating bone homeostasis and contributing to bone pathology (Table 1). Current evidence supports RBPs as potential targets for developing novel therapies to treat various bone diseases. However, research on RBPs in bone pathology is still at an early stage, and establishing RBPs as reliable targets is a work in progress.

**Figure 4:**
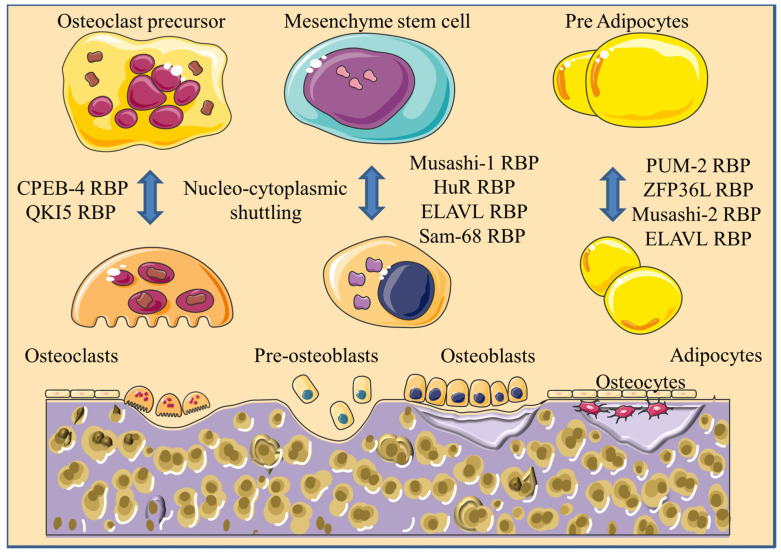
Schematic diagram illustrating nucleocytoplasmic shuttling of RBPs during the formation of osteoblasts, osteoclasts, and adipocytes from precursor cells in the bone microenvironment. CPEB4 and QKI5, which are localized in the cytoplasm of osteoclast precursor cells, are translocated to the nucleus in mature osteoclasts. Musashi-1, HuR, ELAVL, and Sam68, which are localized in the nucleus of MSCs, are translocated to the cytoplasm of preosteoblasts.

Future work may utilize advanced technologies such as CLIP sequencing, CRISPR-Cas9-based gene editing, co-immunoprecipitation, and molecular imaging to further the understanding of RBPs and their mechanisms of action in bone biology and pathophysiology. In particular, the intricate regulatory interactions of RBPs with various RNA molecules, including mRNA, miRNA, lncRNA, and circRNA, warrant comprehensive investigation. These interactions need rigorous validation to mitigate false positives, which could be addressed using techniques such as cellular thermal shift assays and microscale thermophoresis. The dynamic nucleocytoplasmic shuttling of RBPs at various stages of bone diseases could be another focal point of the research (Figure 4).

The aggregation of RBPs is a pathological hallmark of neurodegenerative diseases such as ALS and FTD [63]. In these disease, the RNA-mediated phase transition of RBPs is a key mechanism leading to the deposition of RBP aggregates in degenerating lesions. To date, there have been few reports on RBP aggregation in bone tissues. In future research, molecular dynamics simulations, thermal aggregation assays, protein chromatography, and X-ray scattering techniques can be used to investigate RBP aggregation in bone biology and pathology. Using nomogram models to analyze microarray and RNA-seq data in the public domain may identify new RBP diagnostic markers or therapeutic targets for bone diseases. The interactions between the RBP-RNA network and cell components such as ribosomes, the ubiquitin–proteasome system, and the cytoskeleton can be investigated using genome-wide transcriptome analysis. Overall, a systematic approach to screen and identify altered RBPs in disease-affected bone tissue could help identify novel molecular entities for therapeutic intervention.

**Table 1:** RBPs involved in bone development and diseases.

RBP	Biological Functions in Bone Development and Diseases	References
Musashi-1	Upregulated in bone-marrow-MSCs and bone cells during bone healing and correlates with osteogenic differentiation	[19,20,21]
Musashi-2	Controls osteoblast-adipocyte lineage commitment by suppressing PPARγ	[22]
	Promotes osteoclastogenesis and osteoclast survival through Notch and NF-κB	[39]
ZFP36L1	Suppresses bone-marrow-MSC adipogenic differentiation by directly targeting PPARGC1B and PPARγ-2 mRNAs	[23,29]
	Upregulated in OA chondrocytes and promotes OA by directing targeting the HSP70 family mRNAs	[54]
PUM2	Enhances MSC adipogenesis and inhibits osteogenesis by directly targeting the JAK2 and RUNX2 mRNAs	[24]
PUM1	Protects against chondrogenic loss in OA by directly targeting the TLR4 mRNA	[25]
ELAVL1	Inhibits bone-marrow-MSC osteogenic differentiation by directly targeting ECM-related mRNAs	[26]
BICC1	Promotes osteoblastogenesis and bone mineral deposition via PKD2	[30]
RBM3	Stimulates osteoblastogenesis via ERK	[31]
Sam68	Inhibits osteogenesis and promotes adipogenesis	[32]
	Promotes FLS inflammation, proliferation, migration, and invasion in RA through NF-κB P65	[33]
SAMD4	Promotes osteogenesis and bone development by binding the MIG6 mRNA and inhibits MIG6 protein synthesis	[34]
CPEB4	Promotes osteoclastogenesis downstream of RANKL	[36]
	Promotes macrophage M2 polarization and OS progression	[37]
CPEB1	Promotes OS progression downstream of miR-377-3p	[38]
TTP	Inhibits inflammation and prevents periodontal bone loss	[40]
IMP2	Regulates osteoclast activity and adhesion	[41]
Mex3B	Inhibits osteoclastogenesis by downregulating DCSTAMP	[42]
QKI	Promotes osteoblastogenesis, inhibits osteoclastogenesis, and protects against osteoporosis	[43]
QKI5	Inhibits osteoclastogenesis by downregulating CSF-1R and RANK	[44]
PCBP1	Protects osteoblasts and inhibits ferroptosis induced by high glucose in diabetic osteoporosis	[48]
HuR	Upregulated in OS tissues and cell lines and promotes OS progression by binding the AGO2, HMGA1, and YAP mRNAs	[49,50,51]
	Promotes osteoblastic differentiation and protects against osteoporosis by binding and stabilizing the LRP6 mRNA to activate Wnt	[60]
	Promotes osteoblastic differentiation and protects against osteoporosis by translocation to the cytoplasm upon association with circStag1 to activate Wnt	[61]
Lin28A	Promotes OS cell progression by binding LncRNA MALAT1	[52]
	Promotes osteogenic differentiation by interacting with LncRNA TUG1	[64]
PTBP1	Promotes OS cell viability, proliferation, migration, and invasion by binding ZMIZ1-AS1 to stabilize the ZMIZ1 mRNA	[53]
	Inhibits osteogenic differentiation, enhances adipogenic differentiation, and promotes osteoporosis by interacting with LncRNA SNHGI to upregulate DNMT1	[59]
GNL3	Upregulated in human OA lesions and promotes OA by upregulating IL24 and PTN	[55,56]
TDP-43	Downregulated in human OA lesions and alleviates cartilage degradation and bone remodeling in OA	[57]
SND1	Enhances IL-1β-induced chondrocyte ferroptosis and promotes OA progression by binding and destabilizing the HSPA5 mRNA	[58]
RBFOX2	Its cytoplasmic localization in calcific tendons may contribute to the development of calcific tendinopathy	[62]

## Data Availability

Data sharing does not apply to this article, as no new data were created or analyzed in this study.

## Nomenclature

RBPs	RNA-binding proteins
MSCs	Mesenchymal stem cells
RUNX2	Runt-related transcription factor 2
OSX	Osterix
RANKL	Receptor activator of nuclear factor kappa B ligand
POSTN	Periostin
PPARγ	Peroxisome proliferator activated receptor γ
AA	Aplastic anemia
NF-κB	Nuclear factor κB
TRL-4	Toll-like receptor 4
ELAVL-1	Embryonic lethal abnormal version like 1
ECM	Extracellular matrix
ChREBP	Carbohydrate responsive element binding protein
BICC1	Bicaudal C homolog-1
BMD	Bone mineral density
PKD2	Polycystic kidney disease 2
ERK	Extracellular-signal-regulated kinase
P38	P38 kinase
JNK	C-Jun N-terminal kinase
Sam68	Src associated in mitosis 68 KDa
RA	Rheumatoid arthritis
FLS	Fibroblast like synoviocytes
SAMD4	Sterile alpha motif domain containing protein 4
MIG-6	Mitogen inducible gene 6
TTP	Tristetraprolin
TNFα	Tumor necrosis factor α
IMP2	Insulin like growth factor 2
HCMV	Human cyotomegalovirus
DM	Diabetes mellitus
DMT1	Divalent metal transporter 1
OS	Osteosarcoma
EMT	Epithelial–mesenchymal transition
ZMIZ1-	AS1 Zinc finger MIZ type containing 1 antisense
DNMT	DNA methyltransferase methylation
GNL3	Nuclear GTP binding protein 3
TDP-43	TAR RNA binding protein 43
SND-1	Staphylococcal nuclease domain containing 1
HSPA-5	Heat shock protein family A member 5
OVX	Ovariectomy
RBFOX2	RNA binding protein Fox-1 homolog-2
ALS	Amyotrophic lateral sclerosis
FTD	Frontotemporal dementia
CPEB	Cytoplasmic polyadenylation element binding protein
